# SARS-CoV-2 Infection in Health Workers: Analysis from Verona SIEROEPID Study during the Pre-Vaccination Era

**DOI:** 10.3390/ijerph18126446

**Published:** 2021-06-14

**Authors:** Stefano Porru, Maria Grazia Lourdes Monaco, Angela Carta, Gianluca Spiteri, Marco Parpaiola, Andrea Battaggia, Giulia Galligioni, Beatrice Ferrazzi, Giuliana Lo Cascio, Davide Gibellini, Angelo Peretti, Martina Brutti, Stefano Tardivo, Giovanna Ghirlanda, Giuseppe Verlato, Stefania Gaino, Denise Peserico, Antonella Bassi, Giuseppe Lippi

**Affiliations:** 1 Section of Occupational Medicine, Department of Diagnostics and Public Health, University of Verona, 37134 Verona, Italy; stefano.porru@univr.it (S.P.); angela.carta@univr.it (A.C.); 2 Clinical Unit of Occupational Medicine, University Hospital of Verona, 37134 Verona, Italy; gianluca.spiteri@aovr.veneto.it; 3 Postgraduate School of Occupational Medicine, University of Verona, 37134 Verona, Italy; marco.parpaiola@studenti.univr.it (M.P.); andrea.battaggia@studenti.univr.it (A.B.); giulia.galligioni@studenti.univr.it (G.G.); beatrice.ferrazzi@studenti.univr.it (B.F.); 4 Local Heath Authority of Piacenza, 29121 Piacenza, Italy; g.locascio@ausl.pc.it; 5 Section of Microbiology, Department of Diagnostics and Public Health, University of Verona, 37134 Verona, Italy; davide.gibellini@univr.it; 6 Unit of Microbiology and Virology, University Hospital of Verona, 37134 Verona, Italy; angelo.peretti@aovr.veneto.it (A.P.); martina.brutti@aovr.veneto.it (M.B.); 7 Section of Hygiene, Department of Diagnostics and Public Health, University of Verona, 37134 Verona, Italy; stefano.tardivo@univr.it; 8 Medical Direction, University Hospital of Verona, 37134 Verona, Italy; giovanna.ghirlanda@aovr.veneto.it; 9 Unit of Epidemiology and Medical Statistics, Department of Diagnostics and Public Health, University of Verona, 37134 Verona, Italy; giuseppe.verlato@univr.it; 10 Laboratory of Clinical Chemistry and Hematology, University Hospital of Verona, 37134 Verona, Italy; stefania.gaino@aovr.veneto.it (S.G.); antonella.bassi@aovr.veneto.it (A.B.); giuseppe.lippi@univr.it (G.L.); 11 Section of Clinical Biochemistry, Department of Neurosciences, Biomedicine and Movement Sciences, University of Verona, 37134 Verona, Italy; denise.peserico@univr.it

**Keywords:** SARS-CoV-2 infection, serosurvey, health workers

## Abstract

Background: To report the baseline phase of the SIEROEPID study on SARS-CoV-2 infection seroprevalence among health workers at the University Hospital of Verona, Italy, between spring and fall 2020; to compare performances of several laboratory tests for SARS-CoV-2 antibody detection. Methods: 5299 voluntary health workers were enrolled from 28 April 2020 to 28 July 2020 to assess immunological response to SARS-CoV-2 infection throughout IgM, IgG and IgA serum levels titration by four laboratory tests. Association of antibody titre with several demographic variables, swab tests and performance tests (sensitivity, specificity, and agreement) were statistically analyzed. Results: The overall seroprevalence was 6%, considering either IgG and IgM, and 4.8% considering IgG. Working in COVID-19 Units was not associated with a statistically significant increase in the number of infected workers. Cohen’s kappa of agreement between MaglumiTM and VivaDiagTM was quite good when considering IgG only (Cohen’s kappa = 78.1%, 95% CI 74.0–82.0%), but was lower considering IgM (Cohen’s kappa = 13.3%, 95% CI 7.8–18.7%). Conclusion: The large sample size with high participation (84.7%), the biobank and the longitudinal design were significant achievements, offering a baseline dataset as the benchmark for risk assessment, health surveillance and management of SARS-CoV-2 infection for the hospital workforce, especially considering the ongoing vaccination campaign. Study results support the national regulator guidelines on using swabs for SARS-CoV-2 screening with health workers and using the serological tests to contribute to the epidemiological assessment of the spread of the virus.

## 1. Introduction

The second and third waves of the severe acute respiratory syndrome coronavirus 2 (SARS-CoV-2) pandemic outbreak are still ongoing. As of 9 April 2021, nearly 133 million cases of COVID-19 have been confirmed worldwide, averaging over 2.8 million deaths [1]. As of 9 April 2021, nearly 3.6 million cases have been diagnosed in Italy, approximating 111,000 deaths. In addition, according to the Italian National Health Institute, almost 130,000 health workers were also infected [2].

Many efforts are continuously being made for identifying SARS-CoV-2 positive cases and for epidemiological characterization of the disease spread. Molecular testing enables timely identification of outbreaks, control tracing and containing virus spread, both in the general population and in occupational contexts at higher risk environment, such as healthcare [3,4]. The mean seroprevalence in healthcare workers (HWs) seems to be around 8.7%, with a wide range of values reported in different studies (i.e., from 0% to over 45%) [5]. Seroprevalences mainly vary depending on the study population, sensitivity and specificity of antibody testing, preventive measures, study quality, and the different prevalence of the disease in the general population. In particular, higher seropositivity rates were observed in people working in direct contact with COVID-19 patients, especially within COVID-19 wards [5].

The real-time reverse transcription-polymerase chain reaction (rRT-PCR) on respiratory tract specimens is still currently considered the gold standard for diagnosing SARS-CoV-2 infection. The measurement of anti-SARS-CoV-2 specific antibodies (IgG and IgM) serves as an additional tool for disease detection and disease monitoring in specific circumstances [6], as well as for purposes of epidemiological appraisals and evaluations. The most sensitive and early serological marker entails assessing total antibodies, whose levels start to increase from the second week of symptom onset. Although specific IgM and IgG are detectable as early as the fourth day after symptom onset, higher values usually occur in the second and third week of the illness [7].

These serological tests may contribute to identifying people with previous SARS-CoV-2 infection who have developed an immunological response and assessing the outcome of public health interventions, prioritizing vaccinations, and, most importantly, verifying the effectiveness of vaccination [8,9]. Moreover, large serological mapping of SARS-CoV-2 infection within populations is becoming even more important since the vaccinations campaign began worldwide.

The 27 December 2020 was defined as Europe Vaccine-day, and since that date, each country has embarked on mass vaccination campaigns, prioritizing HWs for their crucial role against the pandemic and their higher risk of being infected.

In this context, serological tests become indispensable for evaluating both efficiency and outcome of the ongoing vaccine campaigns and defining individual risk profiles. Additionally, anti-SARS-CoV-2 antibodies titration can also help assess the spread of new virus variants (such as B.1.1.7, B.1.351 or P1/2), which have spread worldwide, including Italy.

Within the framework of the overall SARS-CoV-2 health and epidemiological surveillance activities conducted at the University Hospital of Verona [4], the so-called SIEROEPID study was designed with the following main aims:

- Investigate the immunological response of HWs to SARS-CoV-2 infection through assessment of serum levels of anti-SARS-CoV-2 IgG, IgM and IgA and their time trends along with different points in time.
- Correlate seroprevalence to socio-demographic and occupational variables, personal habits, clinical data and diagnostic tests for SARS-CoV-2 infection.
- Evaluate the antibody response, to appraise the level of immunological protection, in order to support individual risk assessment, define proper health surveillance protocols as well as lead the decisional process of return to work of the HWs.
- Establish a biobank for further analyses.
- Compare the performances of various laboratory tests regarding sensibility, specificity and accuracy.

The SIEROEPID study was recently expanded to include serial testing of neutralizing antibodies (i.e., anti-SARS-CoV-2 receptor-binding domain immunoglobulins) for HWs adhering to the anti-SARS-CoV-2 vaccination campaign, which commenced at the end of December 2020, with the primary objective of evaluating vaccine effectiveness, safety profiles and the clinical impact of vaccination.

## 2. Materials and Methods

### 2.1. Study Design, Setting and Population

SIEROEPID is an ongoing observational prospective longitudinal survey involving all HWs at the University Hospital of Verona. Voluntary enrolment lasted from 28 April 2020 to 28 July 2020.

### 2.2. Blood Sample Collection, Serological Tests and RT-PCR Test

The HWs were invited to participate, indicating to be present between 7:00 am, and 11:00 am at fasting or having eaten only a light breakfast. After obtaining written informed consent from medical personnel, a serum sample was drawn, shipped within 3 h to the central laboratory, where it was subsequently heat-inactivated at 56 °C for 30 min before analyses. The sample was analyzed with two IgM/IgG tests (provided by the Regional Health Authority of Veneto and University Hospital of Verona):

- MAGLUMI™ 2000 Plus, a chemiluminescent analytical system (CLIA) [10,11]
- VivaDiagTM, COVID-19 IgM/IgG Rapid test, a Lateral-Flow Immunochromatographic Assay (LFIA) [11].

Prima Professional (COVID-19 IgG/IgM Rapid Test, PRIMALab SA), another LFIA, was used as a rapid test for samples taken from 1 to 15 July.

A smaller number of samples were analyzed by an additional test, EuroimmunTM, an enzyme-linked immunosorbent assay (ELISA) that provides semi-quantitative in vitro determination of human antibodies of immunoglobulin classes IgA and IgG against SARS-CoV-2 in serum or EDTA plasma [12,13].

Mass screening with an oro-nasopharyngeal swab was constantly offered from February 2020 for the Verona hospital population. All swabs performed at least seven days before the serological test were considered for this study. Respiratory specimens were tested for SARS-CoV-2 infection by commercial real-time PCR method, Seegene AllplexTM2019-nCoV Assay (Seegene, Seoul, South Korea), according to manufacturer protocols and ISS and CDC guidelines, as specified elsewhere [14].

### 2.3. Communication and Management of Results

According to the study protocol, test results were available to HWs via a dedicated electronic health platform. In the case of the serological test’s positivity, workers were promptly informed, and a swab test was scheduled within 24–48 h to confirm the infection; on a case-by-case basis, possible temporary restriction from work was considered.

### 2.4. Outcomes and Endpoints

HWs’ immunological response to SARS-CoV-2 infection was assessed with IgM, IgG and IgA serum levels; both presented as dichotomous variables (presence/absence of specific antibodies) and continuous quantitative variables (numerical value). IgA data are part of an analysis that will be reported on a companion paper. In addition, the association of antibody titre with several demographic variables (e.g., age, gender, occupation, ward, exposure history) and the swab test results were also addressed. Finally, the comparison between the tests was carried out by assessing both sensitivity, specificity and agreement.

### 2.5. Statistical Analysis

Health workers employed at the University Hospital of Verona were 6258. As a preliminary evaluation had shown an 80% participation rate to serological tests, we anticipated that information on a serological response would be available for about 5000 HWs (6258 × 0.8 = 5006). A previous study had found a 4.0% cumulative incidence of SARS-CoV-2 infection from 28 February to 28 April in the same population [4]. Assuming a slightly higher seroprevalence (5%) in the following trimester, the expected precision of the estimate was 0.6% (95% CI 4.4–5.6%).

The Clopper–Pearson method was used to calculate the 95% confidence interval (CI) of the cumulative incidence of seropositivity to SARS-CoV-2 infection. Fisher’s exact test or the chi-square test for categorical variables evaluated the significance of differences among groups. The strength of association between immunological tests and SARS-CoV-2 infection (assessed by oro-nasopharyngeal swab at least one week before) was evaluated by Cramér’s V. The agreement between different immunological tests was evaluated by Cohen’s kappa coefficient of agreement. Multivariable analysis was performed using a logistic regression model, where seropositivity to SARS-CoV-2 was set as the dependent variable, whilst gender, age, work setting, working in COVID-19 unit, and occupation were the independent variables. Goodness-of-fit was verified by the Hosmer–Lemeshow test. Results were synthesized through the odds ratio (OR).

### 2.6. Ethics

Workers received written information and signed informed consent. The Clinical Experimentation Ethics Committee of Verona and Rovigo approved this study (protocol no. 22851, 23 April 2020).

## 3. Results

### 3.1. Sample Characteristics

From 28 April till 28 July 2020, 5299 HWs were enrolled in the study (84.7% of the target population), whilst 959 HWs declined to participate in the study or did not present after two consecutive invitations. The sample consisted of 28.7% men and 71.3% women, aged 44.1 ± 11.8 (mean ± SD; range 22.3–70.3) years. Nearly 89.3% (*n* = 4734) were HWs dedicated to direct patient care, and 993 (18.7%) worked at COVID-19 Unit Care. Further details are reported in Table 1. Oro-nasopharyngeal swabs, taken at least one week before the serological assessment, were positive in 212 (4.0%) HWs and indeterminate in 126 (2.4%).

### 3.2. Serological Tests

#### 3.2.1. Maglumi Test

When using the Maglumi test, IgM and IgG to SARS-CoV-2 were detected in 109 and 255 HWS, corresponding to 2.1% and 4.8% respectively of the 5299 HWS screened. Overall, 316 HWs (6.0%, 95% Confidence Interval 5.3–6.6%) had either IgM or IgG to SARS-CoV-2. Both IgM and IgG were simultaneously detected in 48 HWs, representing 15.2% of all positive subjects.

#### 3.2.2. VivaDiag LFIA Rapid Test

When using the VivaDiag LFIA rapid test, IgM and IgG to SARS-CoV-2 were detected in 210 and 224 subjects, corresponding to 4.1% and 4.4% of 5080 HWSs screened, respectively. Overall, 247 HWSs (4.9%, 95% CI 4.3–5.5%) had either IgM or IgG to SARS-CoV-2. In addition, both IgM and IgG were simultaneously detected in 187 HWSs, representing the most positive subjects (75.7%).

#### 3.2.3. Prima Professional LFIA Rapid Test

When using the Prima Professional LFIA test, IgM to SARS-CoV-2 was detected in just one subject, and IgG in 19, corresponding to 0.5% and 8.7% of the 218 HWSs screened, respectively. Overall, 19 HWs (8.7%, 95% CI 5.3–13.3%) had either IgM or IgG to SARS-CoV-2. In addition, both IgM and IgG were simultaneously detected in just one HWs, representing 5% of positive subjects.

#### 3.2.4. Euroimmun Anti-SARS-CoV-2 IgA and IgG ELISA Test

A total of 393 high-risk HWs were also simultaneously screened by ELISA test to detect Anti SARS-CoV-2 IgG and IgA. The prevalence of positive IgG was slightly lower with the Euroimmun test (33.8%, *n* = 133) than with the Maglumi test (38.4%, *n* = 151). The agreement between the two tests was fairly good (Cohen’s kappa = 61.3%, 95% CI 53.2–69.4%). Positive IgA was detected in 118 out of 390 (30.3%) with a good agreement with IgG also assessed with Euroimmun (Cohen’s kappa = 72.4%, 95% CI 65.1–79.8%).

### 3.3. Accuracy of Serum IgG Levels vs. Oro-Nasopharyngeal Swab

**Figure 1:**
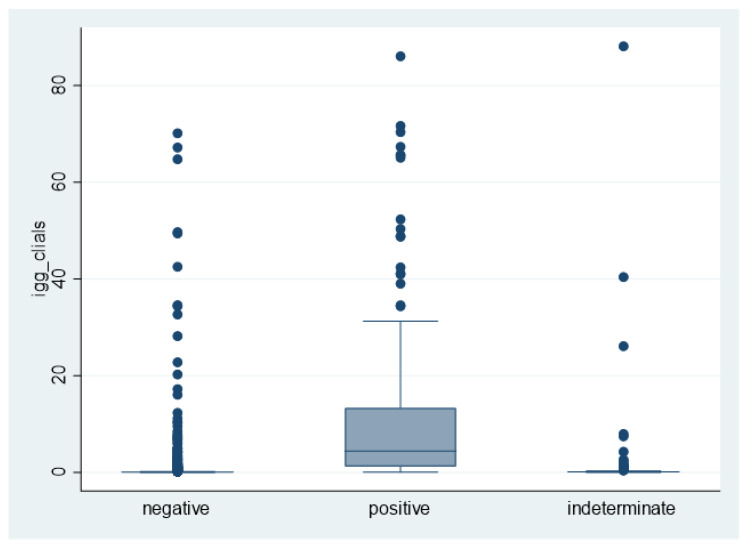
Box-and-whiskers plot of IgG levels, as assessed by MAGLUMI, as a function of the oro-nasopharyngeal swab.

Serum IgG test to SAR-CoV-2, assessed with Maglumi, was positive in 1.7% (82/4961), 10.3% (13/126) and 75.5% (160/212) of HWs with a negative, indeterminate, and positive swab, respectively. Median IgG levels (p25–p75) to SARS-CoV-2 were 0.07 (0.05–0.11), 0.10 (0.07–0.15) or 4.41 (1.18–13.36) kU/l in HWS with negative, indeterminate or positive swab, respectively (Figure 1).

Similar values were obtained when serum IgG levels were assessed by LFIA: an immunological response to SARS-CoV-2 was detected in 1.5% (72/4957), 11.1% (14/126) and 74.1% (157/212) of HWs with a negative, indeterminate and positive swab respectively.

The association between molecular and serological tests (Figure 1) was similar, irrespective of the method used to assess IgG to SARS-CoV-2. Specifically, Cramér’s V was 0.677 for the Maglumi method and 0.682 for the rapid test.

The molecular test displayed a weaker association with IgA than with IgG to SARS-CoV-2, as evaluated by the Euroimmun test. Positive IgG could be detected in 18.8%, 15.0% and 91.9% of subjects with negative, indeterminate and positive swabs, respectively, while positive IgA was found in 20.1%, 15.0% and 72.1%, respectively. Accordingly, Cramér’s V was 0.650 for the IgG-based test decreased to 0.487 for the IgA-based test.

### 3.4. Seropositivity, As Assessed by Maglumi Test, and Exposure to SARS-CoV-2

Nearly two-thirds of Anti-SARS-CoV-2 seroprevalence (*n* = 201) were found in workers with a history of previous close contact with a COVID-19 case. The rate of suspect previous infection was approximately 2% in people who did not report close-contact to COVID-19 cases, increasing to nearly 9% in people referring a previous close-contact, irrespective of whether exposure had occurred at work or elsewhere. Additionally, specific symptoms were highly suggestive of SARS-CoV-2 infection, which could be detected in more than one-fourth of symptomatic subjects, but only in 4–5% of asymptomatic subjects.

The seroprevalence was the highest among technical-administrative staff and the lowest among residents, though the difference was not significantly different. Notably, HWs working in COVID-19 units had nearly the same risk of seropositivity as HWs working in other settings. In multivariable analysis, the risk of previous SARS-CoV-2 infection was not significantly modulated by gender or age (Table 1).

### 3.5. Comparing Maglumi VivaDiag LFIA and Euroimmun ELISA Test

Table 2 summarizes IgM and IgG dosage raw results for each test used.

When considering Maglumi as the gold standard, the rapid test displayed 62.3% (197/316; 95% CI 56.7–67.7%) sensitivity and 98.6% (4913/4982; 95% CI 98.3–98.9%) specificity, respectively. However, Maglumi and the rapid test (VivaDiag) were simultaneously compared, and Cohen’s kappa of the agreement was 65.8% (95% CI 61.3–70.4%). The agreement was quite good when considering IgG only (Cohen’s kappa = 78.1%, 95% CI 74.0–82.0%), but was poor as regards IgM (Cohen’s kappa = 13.3%, 95% CI 7.8–18.7%).

The agreement became very low when considering the most recent kit, adopted in July. Cohen’s kappa decreased to 0.51 (0.35–0.67). Overall, 58.5% of subjects testing positive to Maglumi were negative when using the rapid test. Conversely, only 11% (2/19) of HWs with the positive rapid test were negative to Maglumi (Table 3).

## 4. Discussion

The SIEROPID study was designed about two months after the beginning of the SARS-CoV-2 pandemic. The original objectives (i.e., evaluating SARS-CoV-2 immune response among HWs, appraising SARS-CoV-2 determinants, analyzing tests performance, carrying out a comprehensive epidemiological evaluation) remain important and addressed in this and other papers. However, while the study was performed with blood samples collected, the epidemiological scenario changed, and, most importantly, some COVID-19 vaccines have now become available. Therefore, the study was enriched with additional aims focusing on vaccine effectiveness. This article reports the first phase of the SIEROEPID study results, which concerns the first wave of pandemic spread in Italy between spring and fall 2020.

### 4.1. SARS-CoV-2 Seroprevalence and Determinants

In this study, the overall seroprevalence of SARS-CoV-2 infection was 6%, considering either IgG and IgM, and 4.8% only considering IgG. The literature shows an IgG antiSARS-CoV-2 seroprevalence in Italian HWS ranging from 0.7–1.9% according to researches carried out in Center-Southern Italy [15,16,17], to 4.1–7.4% [3,18,19,20,21] as reported from the surveys in Northern Italy. These differences reflect the epidemiological trend of infection in the Italian population during the first wave, which was more sustained in Northern Italy (including the Veneto Region). Data are similar to those recently reported in Europe (7.7%), higher than in Asia (4.8%) and lower than in the USA (12.4%) [22].

However, while the study was performed with blood samples, the epidemiological scenario changed due to national and local regulators’ containment measures [23]. In fact, in the Veneto Region, the weekly number of subjects with a positive swab increased to about 3000 in mid-March and decreased to 23 cases by around mid-June [24]. Additionally, most importantly, from December 2020 some COVID-19 vaccines have now become available. With regards to the prevalence of active or past SARS-CoV-2 infection among the general population, Guerriero et al. [25] reported data on 1515 asymptomatic subjects, with 0.6% tested positive for SARS-CoV-2 RNA and negative for IgG against SARS-CoV-2, 2.6% tested negative for viral RNA and positive for IgG, and 96.7% tested negative for both indicators.

In two recent reviews, males were more seropositive than females. However, these studies also indicate no statistically significant difference in seroprevalences, according to gender [5,22].

For demographic variables (including gender), no significant associations with Ig positivity could be found in our study. In Italian settings, the Ig rate was found to be significantly higher among males than females in some studies [18,19], whilst no differences were observed among the two groups in others [3,15,21]. Therefore, we put forward the concept that gender may not be a significant determinant of SARS-CoV-2 seropositivity.

As far as the occupational factors are concerned, working in COVID-19 wards was not associated with a statistically significant increase in the number of infected, whilst seroprevalence was found to be relevant among personnel not engaged in direct care as administrative staff. This particular condition was determined by the presence of some clusters of SARS-CoV-2 infection which occurred in the early stages of the first wave, especially for infections consequent to meetings attendance in the presence of health directors and managers involved in the management of the emergency, which in turn operated in close contact with its own administrative staff. To this end, De Carlo et al. [17] found a higher proportion of positive subjects in the intermediate-risk group (IgG 1.2%, IgM 1.1%) and low-risk group (1.3%) rather than in the high-risk group (IgG 0.7%, IgM 0.9%). As expected, with a progressive variation of the epidemiological burden of SARS-CoV-2 infections over time and the different occupational settings, other studies have shown other evidence.

Finally, our data suggest that the current SARS-CoV-2 serology may be of limited clinical utility for diagnosing acute infection. All HWs workers that had IgG or IgM positivity without previous positive swab were asymptomatic, and the RT-PCR test carried out in the following 24/48 h was negative, in keeping with the currently available literature data [26]. It is conceivable that these subjects had previous contact with the virus in an asymptomatic or symptomatic form but that they have not been swab tested, because they were initially placed in quarantine. Furthermore, it cannot be ruled out that some may have contracted the infection before the pandemic’s start and that this could have been confused with seasonal flu. Finally, in cases of positivity to the serological test just above the cut-off, the data could fall within the percentage of non-specificity of the test. According to the study protocol, all HWs testing positive for IgM and IgG were offered to undergo swab testing to diagnose an acute SARS-CoV-2 infection; none of these tests resulted positive.

### 4.2. Test Performance Comparison

The SIEROEPID study has also addressed and compared with three SARS-CoV-2 antibody assays. When the study began, a gold standard for anti-SARS-CoV-2 Ig detection was not yet available. Therefore, a test comparison was made considering Maglumi as the gold standard. In the first analysis, the rapid test displayed lower sensitivity (62.3%) and good specificity (98.6%) [16]. Limited to IgG detection, Maglumi demonstrated a good agreement with ELISA (Cohen’s Kappa = 0.61) and even more with the LFIA test (Cohen’s Kappa = 0.78). Conversely, low concordance was found between CLIA and LFIA concerning IgM tests (Cohen’s Kappa = 0.13). This is not surprising since the biological role of these immunoglobulins has been seriously disputed, as well as their clinical significance for diagnosing an acute SARS-CoV-2 infection [27]. Therefore, our findings would reinforce the concept that measuring anti-SARS-CoV-2 IgM is probably needless, if not even misleading.

Comparing tests was one of our original objectives, as many immunoassays with different claimed performances have become available. Although the clinical significance of antibody testing for diagnosing SARS-CoV-2 infections has gradually decreased, test validation remains an important aspect, especially considering the different socio-demographic and occupational contexts that can be explored through seroprevalence surveys. Whilst laboratory-based serological tests have a great value in occupational high-risk settings investigation, the possibility of using rapid serological tests should not be discounted. This would mainly concern decentralized settings encompassing the need to carry out a high volume of tests in a short time (e.g., before vaccination), thus taking profit from simpler and possibly cheaper approaches. Developing countries are other ideal settings for rapid serological tests, since, in these areas, SARS-CoV-2 infections continue to spread, causing a huge number of deaths, as well as during emergencies of public health (e.g., war zones), where decentralized testing is virtually the only available option.

The good agreement between CLIA and LFIA IgG titration suggests the need to continue the research in this field to improve test performance.

### 4.3. Further Issues

The study included a considerable number of participants. Therefore, seroepidemiology of the SARS-CoV-2 infection could cover nearly the entire hospital. This is a significant achievement because the baseline dataset is essential for decision-making and managing further scenarios.

Since 27 December 2020, the HWs of the University Hospital of Verona were offered to undergo SARS-CoV-2 vaccination; as of 8 April 2021, over 5600 HWS completed the two-dose vaccination course, and almost 6000 received their first dose. For 12 months after vaccination, antibody titration will be monitored.

Collecting a baseline value to humoral immunity will make it possible to study the kinetics of the immunological response and identify personal or occupational factors that might influence such response since this population is accurately characterized and followed up through ongoing risk assessment and health surveillance programs. Moreover, knowledge of previous antibody titre deriving from contact with the virus may guide vaccines’ administration according to international and national guidelines and represents one key element of knowledge to achieving herd immunity [22]. Therefore, the information garnered from this study may become a benchmark, allowing to obtain baseline data for future risk assessment and management of SARS-CoV-2 infection both in individual HWs as well as the entire hospital workforce. This is particularly important in the light of the spread of new SARS-CoV-2 variants and the need for epidemiological and virologic investigations designed to assess transmissibility, the severity of the disease, risk of reinfection, quality, amount and duration of antibody response to these new clinically threatening variants [28].

Another important issue concerns the ongoing nationwide vaccination coverage, which, on 10 April 2021, was completed in about 3.8 million people [29]. Since vaccination is voluntary, not all health HWs are or will be vaccinated, whilst vaccination of fragile people has just recently begun. Therefore, with such a dramatic evolution of the pandemic outbreak, immune status monitoring is unavoidable for better protection and use of the health care force.

### 4.4. Strengths and Limitations

The significant sample size, the monocentric design, the high participation rate, the construction of a reliable baseline dataset, the determination of various indicators, the comparison of various tests, the thorough characterization of the study population with regular follow-up are the main strengths of this study.

This is even more important because the work setting belongs to an Italian area significantly affected by the COVID-19 pandemic, such as the Veneto Region.

The possible limitations are the tumultuous development of the epidemiological scenarios, the dramatic technological improvements, and the rapid growth of scientific knowledge on SARS-CoV-2 infection, which make it difficult to interpret data collected only a few months before. Authors should discuss the results and how they can be interpreted from previous studies and the working hypotheses. The findings and their implications should be discussed in the broadest context possible. Future research directions may also be highlighted.

## 5. Conclusions

In conclusion, this research carried out in a significant and well-characterized sample size showed a significant seroprevalence of SARS-CoV-2 infection in HWs in a largely affected area in Northern Italy, consistent with studies carried out in similar contexts, and higher than the general population residing in the same area. The study offers relevant data on serologic test performance, especially considering the current lack of a gold standard, and confirms their role in the SARS-CoV-2 seroprevalence assessment, especially in higher exposure environments. Moreover, the longitudinal design will enable us to study the time trends of the seroprevalence and, together with the biobank, to target proper risk assessment, health surveillance and health promotion programs, especially considering the ongoing vaccination campaign, now witnessing a very high participation rate in our study population, which will also allow us to monitor the compliance and effectiveness of immunization versus SARS-CoV-2.

## Figures and Tables

**Table 1:** Previous SARS-CoV-2 infection, as detected by Maglumi test (IgG), in 5299 workers of Verona University Hospital, as a function of main demographic and occupational characteristics. A multivariable logistic model derived odds ratio (OR) with the corresponding 95% confidence intervals (95% CI) and *p* values.

	N	Maglumi Positive Test (IgG)	*p* Value *	OR (95% CI)	*p* Value *
Gender			0.076	0.72 (0.54–0.96) F vs. M	0.028
Male	1520	86 (5.7%)			
Female	3779	169 (4.5%)			
Age (years)			0.273		0.324
22–29	988	45 (4.6%)		1 (reference)	
30–39	989	39 (3.9%)		0.76 (0.47–1.22)	
40–49	1266	58 (4.6%)		0.84 (0.51–1.38)	
50–59	1646	95 (5.8%)		1.07 (0.66–1.73)	
60–70	410	18 (4.4%)		0.75 (0.39–1.45)	
Working in COVID-19 unit			0.742		0.551
No	4306	205 (4.8%)		1 (reference)	
Yes	993	50 (5.0%)		1.11 (0.79–1.54)	
Profession			0.356		0.458
Physician	746	37 (5.0%)		1.02 (0.64–1.60)	
Nurse	1919	97 (5.1%)		1.11 (0.78–1.60)	
Other health professionals	1085	50 (4.6%)		1 (reference)	
Resident	960	*36 (3.8%)*		0.74 (0.42–1.28)	
Technical-administrative staff	565	33 (5.8%)		1.24 (0.79–1.94)	
Other	24	2 (8.3%)			
Type of contact			**<0.001**		
No close contact	3349	74 (2.2%)			
Close contact not at work	301	29 (9.6%)			
Close contact at work	1649	152 (9.2%)			
Suggestive symptoms			**<0.001**		
No	4329	150 (3.5%)			
Yes	366	95 (26.0%)			
Unknown	604	10 (1.7%)			

* Bold values denote statistical significance at the *p* < 0.05 level.

**Table 2:** Seropositivity percentage and results for each test used in the study.

	IgM Seropositivity	IgG Seropositivity	IgM/IgG Seropositivity
Maglumi	109/5299 (2.1%)	255/5299 (4.8%)	316/5299 (6.0%)
VivaDiag^TM^	210/5080 (4.1%)	224/5077 (4.4%)	247/5080 (4.9%)
Prima Professional	1/218 (0.5%)	19/218 (8.7%)	19/218 (8.7%)
EuroImmun^TM^	----	133/393 (33.8%)	---

**Table 3:** Cohen’s kappa of agreement between different serum tests, as a function of the lot used. IgG and IgA were assessed in a subgroup of 393 high-risk health workers by Euroimmun test.

	Total (*n* = 5299)	Before 6 of June (*n* = 4255)	6–30 June (*n* = 826)	July (*n* = 218)
Maglumi vs Rapid test	0.66 (0.61–0.70)	0.71 (0.67–0.76)	0.41 (0.27–0.56)	0.51 (0.35–0.67)
IgM Maglumi vs Rapid test	0.13 (0.08–0.19)	0.18 (0.12–0.24)	0	−0.01 (−0.03–0.01)
IgG Maglumi vs Rapid test	0.78 (0.74–0.82)	0.79 (0.75–0.84)	0.73 (0.57–0.88)	0.73 (0.58–0.88)
	**Total (*n* = 390)**	**Before 6 June** **(*n* = 276)**	**After 6 June** **(*n* = 114)**	
IgG_Maglumi vs IgG Euroimmune^TM^	0.61 (0.53–0.69)	---	---	
vs IgA Euroimmune^TM^	0.72 (0.65–0.80)	---	---	

## Data Availability

The datasets generated during the current study are not publicly available because they contain sensitive data to be treated under data protection laws and regulations. Appropriate forms of data sharing can be arranged after reasonable request to the PI.
